# The social informatics of knowledge

**DOI:** 10.1002/asi.24205

**Published:** 2019-03-05

**Authors:** Eric T. Meyer, Kalpana Shankar, Matthew Willis, Sarika Sharma, Steve Sawyer

**Affiliations:** ^1^ School of Information, University of Texas at Austin 1616 Guadalupe St. Ste. 5.202, Austin, TX 78705 USA; ^2^ Oxford Internet Institute, University of Oxford 1 St Giles, Oxford UK OX1 3JS UK; ^3^ School of Information & Communication Studies University College Dublin, Newman Building, Belfield Dublin 4 Ireland; ^4^ The School of Information Studies, Syracuse University 337 Hinds Hall, Syracuse New York 13244 USA; ^5^ The School of Information Studies, Syracuse University 344 Hinds Hall, Syracuse New York 13244 USA

## Abstract

In the Introduction to this special issue on the Social Informatics of Knowledge, the editors of the issue reflect on the history of the term “social informatics” and how the articles in this issue both reflect and depart from the original concept. We examine how social informatics researchers have studied knowledge, computerization, and the workplace, and how all of those have evolved over time. We describe the process by which articles were included, how they help us understand the field of social informatics scholarship today, and reflect briefly on what the future of the field holds.

## Introduction

In this special issue of the *Journal of the Association for Information Science and Technology*, the editors and authors explore *The Social Informatics of Knowledge*. Our collective task is to advance the concepts, methods, and theories that support the social informatics perspective. Within the broad space of socio‐technical theories and perspectives, we see social informatics as the study of the interdependencies among people, digital technologies, and their contexts of use (Sawyer & Jarrahi, 2014). Through this lens, scholars can understand a wide variety of topics linked by a recognition of the “integration of information and communication technologies into organizations … [which has] now spread from organizations … [into] people's social lives” (Fichman & Rosenbaum, 2014, p. x). Rob Kling's efforts to define and advocate for social informatics pointed out at the time that in research on socio‐technical models of Information and Communication Technologies (ICT) in society “…knowledge and expertise are inherently tacit/implicit…” (Kling, 2000, p. 220) as opposed to explicit: all too often, the processes of knowledge generation and discovery are hidden behind technology or within a technological black box.

In the intervening 20 years, there has obviously been considerable research on the topic of knowledge in a variety of outlets (see Hislop, 2013, for a comprehensive review). This said, much of this work still focuses on specific practices of knowledge management and are often constrained to the realms of formal organizations (Grant, 2011) instead of the broader socio‐technical questions of how knowledge practices are embedded within and enabled by technical systems that may or may not be part of any one organization. Our goal in this issue is to push the field of social informatics forward by emphasizing the latter, while including methodological and conceptual advances in the former.

This special issue had its start via a call for articles for the Social Informatics Special Interest Group (SIG‐SI) preconference symposium held at the 2017 Annual Meeting of the Association for Information Science and Technology (ASIS&T). We explained in the call our intent to treat the symposium as a space to work with interested authors to extend their conference papers for possible inclusion in this special issue. However, we also invited potential authors who did not attend the workshop to submit; this issue includes articles both from people who were at the workshop and from those who were not. Most of the authors attending the symposium chose to submit their articles for this special issue; with feedback from the symposium attendees and the special issue editors' input, authors expanded their articles and submitted them for review. Some of those have been selected for inclusion here through this journal's normal review processes.

Our call for this issue indicated that we were seeking submissions that could help extend our understanding of how we can better explain knowledge practices by looking at the connections between people and technologies, which Meyer (2014) has elsewhere called “examining the hyphen” in the socio‐technical sphere. Meyer argues that thinking of the hyphen in the compound word “socio‐technical” as a locus of interest allows social informatics scholars to approach research questions without a priori planning to foreground either the social aspects of a configuration or its technological components:

Inherent in the act of examining the hyphen … is a balanced view toward the relative importance of the social and the technical aspects of any given socio‐technical construct. This is important because many of the alternative frameworks for understanding the socio‐technical world rely on *a priori* assumptions, either stated or unstated, that either social considerations or technical considerations are of primary importance (Meyer, 2014, p. 58).

Thus, as social informatics scholars, we do not assume the automatic primacy of social considerations in all situations, but neither are we technological determinists who see technologies “causing” people to behave in certain ways. People and the technologies they use are “co‐constitutive, and this complex interrelationship makes any assumption of causality problematic” (Warschauer, 2002, p. 4). To this end, we encouraged all potential authors to examine Kling's foundational article on the nature of the entanglement between the social and the technical in which he wrote that social informatics is “the interdisciplinary study of the design, uses and consequences of information technologies that takes into account their interaction with institutional and cultural contexts” (Kling, 1999, 2007, p. 205).

## Social Informatics and Knowledge

In order to understand the context of this special issue, it would help readers new to social informatics to understand some brief background about social informatics, its relation to the concept of knowledge, and its origins focused on computerization of workplaces and organizations. We are not going to provide a complete history of the development of social informatics as a concept (for that, see Berleur, Berleur, Nurminen, & Impagliazzo, 2006; Davenport, 2005; Elliott & Kraemer, 2008; Fichman & Rosenbaum, 2014; Kling, Rosenbaum, & Sawyer, 2005; Sawyer & Eschenfelder, 2002; Sawyer & Jarrahi, 2014), but instead we will focus on a few examples that help to understand the social informatics perspective in the context of computing and the changing (and unchanging) aspects of knowledge.

Some of the initial studies and theorizing over four decades ago that advanced what would later come to be called social informatics were done by studying knowledge work within formal organizations, and for a very simple reason: in the 1970s and 1980s, that was where computing could be found. For example, early work such as that by Danziger, Dutton, Kling, and Kraemer (1982) sought to puncture the then‐dominant technopositivist narrative that computing would somehow magically fix the biases in government by the very presence of automated systems, instead arguing that computing reinforced bias and structures of control in the organizations they studied. The utopianist view of computing as a magic bullet for organizational problems has been persistent for decades, and early work such as this was motivated by a desire to better understand the complex nature of computing in organizations. Of course, this was not only an issue in early computing: looking at similar government organizations trying to exploit the latest wave of computerization (namely, “big data”) over 30 years later, Clarke and Margetts (2014) found that “governments have been slow to capitalize on the potential of big data, while the largest data they do collect remain ‘closed’ and under‐exploited” (p. 393) even while the rhetoric of computing as a potential technological savior for governmental problems persists.

Long before the term social informatics was proposed as a way to focus studies of socio‐technical systems, Kling and Scacchi (1982) proposed what they called a “web model of computing” in which they focused on how computing resources were the result of activities and decisions by a network of producers and consumers. Knowledge was at the heart of what Kling and Scacchi were trying to understand, although at the time much of the focus was on trying to understand the knowledge required to make computer systems function at all. In their article, they describe a hypothetical organization, *Audiola,* in which information produced “by the computer” in practice requires a cadre of specialized clerks, data processors, programmers, engineers, managers, and many more in a “production lattice” of people and machines that together form “the computer system” (pp. 20–21). A decade later, Orlikowski (1993) would pick up a similar theme when she described the implementation of what was then called groupware in an organization, noting that “groupware [software] on its own is unlikely to engender collaboration” (p. 237) and will instead be used to control access to knowledge along existing organizational lines of power and influence. These organizational approaches are still valuable (see, for instance, Auernhammer & Hall, 2014, and their study of knowledge creation and innovation in a German manufacturing firm), but this early focus on organizational settings has been expanded in recent years.

These early studies, of course, predate the widespread public adoption of the Internet in the mid‐1990s and the huge growth of the Internet as a contributor to knowledge as measured by its prominence in the scholarly literature (see Meyer, Schroeder, & Cowls, 2016). The Internet and its ability to allow the distributed and shared production of knowledge has contributed not just to the scale and scope of research using digital materials, but has also reconfigured the ways that knowledge is created across disciplines (Meyer & Schroeder, 2009, 2015).

In this era of distributed knowledge, social informatics has been used as a lens to understand many aspects of knowledge production, distribution, and use in the Internet era. For instance, Serenko, Ruhi, and Cocosila (2007) used a social informatics lens to focus on the ways that intelligent agents on the web had the potential for unintended consequences: reconfiguring work, eroding trust in technology, reducing privacy, and creating social detachment. A decade later, intelligent agents (“bots”) and their ability to create “computational propaganda [have] recently exploded into public consciousness … [and are] both a social and technical phenomenon” (Bolsover & Howard, 2017, p. 273). Social informatics and the willingness to grapple with potential unintended consequences sets this perspective apart from some of the less critical literature on technology innovation.

Social informatics approaches also have been used to understand how online and offline knowledge and communication spaces can be conceptualized using social informatics approaches, such as Socio‐Technical Interaction Networks (STINs) (Taylor‐Smith & Smith, 2018). STINs have also been used to understand how stakeholders access knowledge in library collections (Waugh, Hamner, Klein, & Brannon, 2015), how historians create geographical knowledge (Suri, 2011), and how distributed learning can only be understood as a complex interplay among people, technologies, practices, and learning artifacts (Walker & Creanor, 2009). These and other examples are geared toward understanding the complex nature of knowledge creation in the digital era.

It is important to avoid technological determinism, but also to avoid a stance of technological exceptionalism, assuming that everything is fundamentally changed when modern technologies are applied. Recall that technology and society co‐constitute each other. Thus, technology can also be shaped to work in ways consistent with earlier eras, even when the scale, scope, and outward appearance are radically different. One of the knowledge projects of the modern web that was never supposed to have worked but works anyway is Wikipedia. Ford (2015) describes in detail the processes of creating facts in the Wikipedia ecosystem, including the challenge of reacting to fast‐changing events such as the 2011 Egyptian Revolution. Ford concludes that “Wikipedia has become authoritative by a process of reaffirming the authority of traditional experts and in doing so Wikipedia has signaled the rise of new centers of expertise” (p. 251). But before we assume that everything about Wikipedia is de novo, consider the following quote:

In editorial methods and procedures … [it] represents a kind of complex journalism. Its system of continuous revision … requires constant scrutiny of its contents and a steady watchfulness on world events necessitating textual alterations, and makes imperative keeping its information as up to date as is possible in … forty‐one thousand articles comprising more than forty million words (Kogan, 1958, p. 283).

The eagle‐eyed reader will note the date of that quotation, and avoid being lulled into thinking that quote describes Wikipedia. It actually refers to *Encyclopedia Britannica*'s operations over six decades ago. The scale and scope have changed (English Wikipedia is made up of over 5 million articles comprising more than 3.6 billion words according to Wikipedia [http://Wikipedia.org, 2019]), but some of the behaviors and challenges of creating knowledge persist over time. This is why enhancing our fundamental understanding of how people interact with information and technology and use those interactions to create knowledge go beyond the specifics of any given technology or specific socio‐technical configuration. Technology will continue to change, some innovations will require more radical reconfigurations than others, but the social and behavioral processes that form the “socio” side of the socio‐technical configurations are not just affected by these changes, but affect the technologies in turn. Understanding this entanglement of human interaction and technology is at the heart of social informatics. There is not a single way or one‐size‐fits‐all model to look at this relationship; rather, social informatics provides the framework for understanding this entanglement. It leaves the door open for the researcher to hone in on the varying modalities of the social from individuals, groups, organizations, and institutions. It enables the examination of social relations, social practices, and social constructs (Agre & Schuler, 1997) that define human interaction like trust, negotiation, respect, power, hierarchy, gender, race, identity, and other elements of what constitutes the “socio” in socio‐technical.

Computerization has moved beyond work: The rapid and ubiquitous uptake of the Internet, the growth of mobile phone access to the Internet, the explosion of digital content and broadening of who is able to generate content, and the expanding of technology generally in the past 30 years overwhelms our ability to understand, much less assess, what this means for the reshaping of social relations—be they families, communities, friends, workplaces, or social spaces—and what are the new “webs of computing” that are mutually constituted with these reshaped social relations. Socio‐technical configurations that were limited to work settings during the late 1980s are now commonly experienced by young children. Thus, the socio‐technical systems and therefore the interactions (or hyphens) we study are ever‐changing. The boundaries between organizations and individuals are shifting, blurring, and being reconfigured, and the socio‐technical interaction networks (Kling, McKim, & King, 2003; Meyer, 2006) that emerge to allow us to operate within those reconfigured settings are changing.

But while the topics and settings that social informatics scholars study have shifted over the decades, many fundamental insights remain true throughout: That unintended consequences are unavoidable (and may overshadow the expected or intended consequences); that some social groups will benefit more than others from the uses of digital technologies (effects are always unequal); that there are multiple effects from the take‐up and uses of new digital technologies; and that usage and effects are tied to the socio‐technical practices of design and use, situated in specific contexts. So, while uses of new ICTs may be understandable broadly, it is the detailed understanding of uses, effects, adaptations, and reconfigured relations that provide insight on change.

## In This Issue

In this issue, we showcase eight articles, plus this introductory essay by the editors. In these articles, we are in an era where computers have become ubiquitous, and contemporary authors have moved toward “understanding the hyphen” in socio‐technical configurations. By looking at these dynamics in a number of different knowledge settings, we can start to see what the ramifications are for theory and methods of analysis.

As we saw above, early social informatics research tended to focus on the workplace and within formal organizations. Social informatics researchers still study computerization and work, but in the intervening decades the nature of work has changed fundamentally. Computerization and its miniaturization, as well as the Internet, have made new kinds of work possible—mobile work, platform work, large‐scale distributed teamwork, which in turn have pushed for new kinds of computing resources. Work takes place both everywhere and nowhere. It is also increasingly precarious.

Many knowledge workers (either by choice or chance) are no longer located in an office, nor do they work for one organization. They must create personal ecologies of resources, technologies, and people that make their work possible wherever they are while still allowing them to take advantage of their relative freedom from place to travel and explore. Jarrahi, Philips, Sutherland, Sawyer, and Erickson (2019) explore this phenomenon with their article on knowledge practices of digital nomads who, as they write, have “escaped the traditional office work environment by engaging in digital work and by drawing on digital technologies. Digital nomads are unified most strongly by their motivation for living nomadically, which is, in almost every case, a desire for travel and a sense of adventure” [page 315]. For these individuals, the “hyphen” is the personal knowledge management that entails negotiating assemblages of portable devices, distant platforms, and online social networks, making them more similar to their peers rather than being defined by the company for which they work.

Khazraee's article (2019) looks at a very different kind of work and worker that are both highly place‐bound: a Turkish archeological project. Laws in Turkey do not now allow artifacts to be removed for study, which necessitates the collaboration of laboratories and dig sites as well a large cast of workers, including archeologists, database managers, technicians, and administrators, all with different languages, practices, and tasks. The legal framework in which the project Khazaee discusses influences the overall project, which in turn affects the technology and practices of individuals and groups of workers. Likewise, Ward and Given's article (2019) looks at large‐scale team collaborations but includes a new wrinkle with respect to specificity of context and the frictions that are introduced: economic, cultural, and linguistic differences among agricultural teams based in Australia and Lao People's Democratic Republic collaborating together (in English, the native language of the Australian team). Ward and Given articulate the need for evaluating information and communication technologies to be used on such projects and argue for a culturally aware approach to integrating ICTs into projects.

Research and development in computerization over the last few decades, much of it documented and analyzed by social informatics researchers, have precipitated massive change in artificial intelligence and robotics; as a result, humans are now not the only “knowledge workers.” Artificial intelligence (AI) and robots are replacing certain kinds of human knowledge work while supplementing or augmenting others, depending on the organizational context. Beyond the cognitive and organizational dimensions of such work, Pee, Pan, and Cui's article (2019) explores new kinds of frictions between humans and their machines as they “work” together in Chinese hospital settings. From a perspective that examines this kind of knowledge work as forms of embodiment, Pee, Pan, and Cui argue that AI and robots (and their physical form) will have an increasingly important role in shaping the “hyphen.”

Social informatics has traditionally assumed a positive stance toward knowledge work: that it is deployed for positive effect, and that human actors engage in knowledge work to constructive ends. This is not always the case. McCoy and Rosenbaum tackle this issue in their article on the role of decision support systems in higher education (2019). In their ongoing efforts to rationalize and quantify that maddeningly unquantifiable organization, academia, many institutions have developed “dashboards” that purport to increase transparency and accountability in institutional decision‐making. In their article, the authors detail the many practices of the users of these systems that render the dashboards useless for the very kinds of decision‐making that they are designed to support.

Shen, Li, Sun, Chen, and Wang's article (2019) pushes this theme forward: that socially deviant information practices such as knowledge withholding and hoarding are equally valid and viable, especially in online interactions where relationships among the users are loose. Sun and colleagues examine such interactions in a Chinese social network and online question answering and discussion community and raise questions about the transactional costs of knowledge sharing, costs that have seldom been discussed in the social informatics literature.

The last two articles turn to the future: exploring new theoretical and topical framings for social informatics research. Fonseca, Marcinkowski, and Davis' article (2019) is a theoretical inquiry, building on data work and geographical information systems (GIS) to theorize an approach to exploring Big Data and its role in knowledge construction as a “cyber‐human system.” The last article turns the social informatics of knowledge on its head as it lays out a provocative area for social informatics research: ignorance. Greyson's exploration (2019) of the subject argues that although the study of ignorance (“agnotology”) is nascent in information science, the construction and maintenance of ignorance as a socio‐technical process can open up new topics and avenues of inquiry. While methodologically challenging, the study of ignorance as a socio‐technical practice has implications for the study of bias in design and algorithms, the spreading of misinformation and disinformation in social media, and similar current topics.

## Conclusion

Twenty years ago to the month this piece was written, Rob Kling asked: “What is social informatics and why does it matter?” (1999, 2007, p. 205). His response to the first half of the question he posed is: “[T]he interdisciplinary study of the design, uses and consequences of information technologies that takes into account their interaction with institutional and cultural contexts” (Kling, 2007, p. 205). When Kling wrote this piece, these technologies were almost all large‐scale information systems and those institutional and cultural contexts were always the workplace. The people in these analyses were “knowledge workers” and we knew very little, if anything, about them beyond that. As these articles demonstrate, the workplace continues to be a topic of great interest to social informatics, but the character of the workplace, computing technology, and the worker have all changed. Furthermore, as computing technology has evolved it has become an essential and ubiquitous part of people's lives well beyond work. The hyphen in “socio‐technical” has become blurry; there is no longer either one or the other, but both. In short, the answer to the latter half of Kling's question two decades hence is that social informatics matters now more than ever.
